# Target the Muscle, Not the Wrinkle: Botulinum Neurotoxin in Facial Anatomy

**DOI:** 10.1111/jocd.71090

**Published:** 2026-07-31

**Authors:** Kyu‐Ho Yi, Kim Hongeok

**Affiliations:** ^1^ You and I Clinic Seoul South Korea; ^2^ VOS Dermatology Clinic Seoul South Korea

**Keywords:** botulinum toxin, bunny lines, crow's feet, injection anatomy, lateral canthal lines, lateral canthus, orbicularis oculi, ultrasonography

## 1 Introduction

The question of whether botulinum toxin should be injected at the lateral canthus or more laterally into the orbicularis oculi muscle (OOc) is not a minor point of technique. It is a conceptual question about what an aesthetic injector believes the target is.

The visible wrinkle is the endpoint of a dynamic process; it is not necessarily the site where that process should be interrupted. A lateral canthal line is made visible by repeated folding of thin periorbital skin over a contracting sphincteric muscle, by tension from the lateral canthal tendon and orbital rim, and by the way smiling or squinting recruits neighboring midface muscles. Therefore, the most clinically relevant target is not simply the crease. It is the active motor unit and the vector of contraction that produces the crease.

This distinction matters because the lateral canthus is an anatomically crowded region. The eyelid margin, palpebral orbicularis, orbital orbicularis, canthal tendon, orbital septum, lacrimal gland region, venous structures, and zygomatic smile elevators all sit within a small treatment field. An injection that is too close to the eye, too medial, too deep, or too inferior may still be inside the area called “crow's feet,” but it can be outside the ideal muscle target. Conversely, a point that seems slightly lateral to the visible crease may be a better target if it lies in the thicker, contracting lateral orbital orbicularis oculi. The practical message is simple: do not inject the wrinkle; interpret the wrinkle.

Regulatory labeling reflects this principle. The current United States prescribing information for BOTOX Cosmetic describes injections for lateral canthal lines into the lateral orbicularis oculi muscle, with the needle oriented away from the eye, using three sites per side, and with the first site approximately 1.5–2.0 cm temporal to the lateral canthus and just temporal to the orbital rim [1]. These directions should not be copied uncritically across all products, countries, and patients, because botulinum toxin preparations are not interchangeable unit for unit. Nevertheless, the anatomy behind the instruction is clinically important: the landmark is the lateral canthus, but the target is the lateral orbicularis oculi.

The observation that the lateral orbicularis oculi can be thicker is supported by recent anatomical review and ultrasonographic work [2, 3]. Piao et al. used ultrasonography in healthy Korean volunteers and measured the orbicularis oculi muscle at serial lateral canthal landmarks. Their reported mean muscle thickness increased from 0.7 ± 0.3 mm at point A to 1.1 ± 0.3 mm at point B and 1.2 ± 0.3 mm at point C; the corresponding depths from the skin surface were 2.0 ± 0.4 mm, 2.8 ± 0.4 mm, and 3.1 ± 0.5 mm, respectively [3]. The most accurate clinical phrasing is that the more lateral measured portion at the lateral canthal level was thicker than the more medial measured segment, not that the entire lateral orbicularis oculi is uniformly thicker than the entire medial orbicularis oculi. The line may begin at the canthal corner, but the muscle belly and the useful treatment field often extend lateral to the bony orbital rim. This is similar to the lesson emerging from anatomical studies of bunny lines: the crease may be seen in one location, while the responsible muscular borders lie around it [4]. In both regions, line‐chasing may be less anatomically rational than muscle targeting.

**FIGURE 1:**
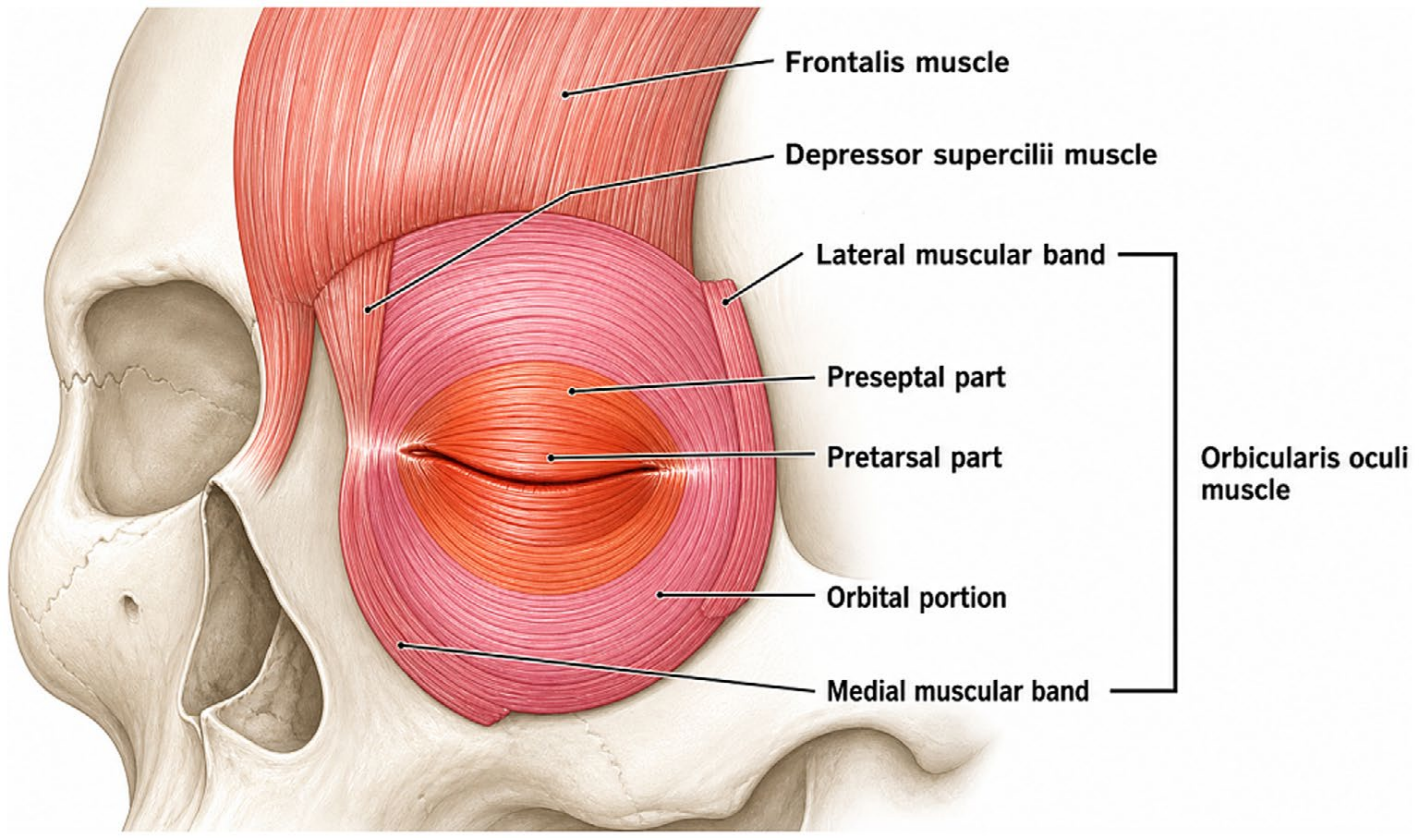
Target the muscle field, not the wrinkle. Schematic anatomy of the orbicularis oculi muscle and its relationship to visible lateral canthal lines.

**FIGURE 2:**
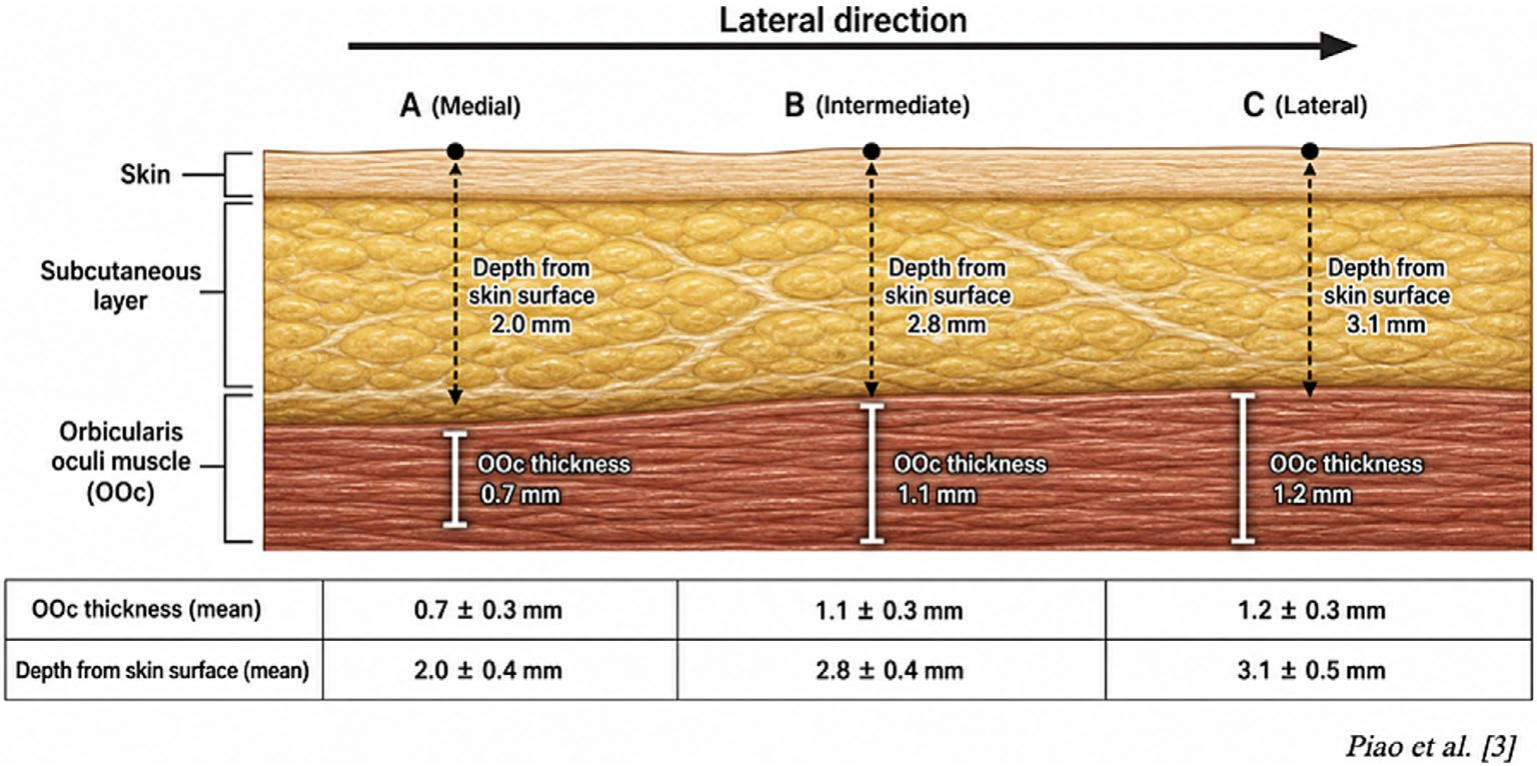
Ultrasonographic thickness gradient of the lateral orbicularis oculi muscle (OOc). Based on the ultrasound study by Piao et al. [3], the measured OOc thickness increased from point A to point C: 0.7 ± 0.3 mm at A, 1.1 ± 0.3 mm at B, and 1.2 ± 0.3 mm at C. The corresponding depths from skin surface were 2.0 ± 0.4 mm, 2.8 ± 0.4 mm, and 3.1 ± 0.5 mm. In precise wording, the more lateral measured portion at the lateral canthal level was thicker than the more medial measured portion, rather than the entire lateral OOc being uniformly thicker than the entire medial OOc. Image regenerated in courtesy of Prof. Hee Jin Kim.

The muscle‐field concept and the ultrasonographic thickness gradient are summarized in Figures 1 and 2, while the principle of avoiding surface‐pattern‐only interpretation is further supported by Rams et al. [5].

## 2 What Creates the Lateral Canthal Wrinkle?

The orbicularis oculi is not a flat sheet of uniform muscle. It is a circumferential sphincter with an orbital portion, palpebral portion, medial band, lateral band, and patient‐specific attachments to adjacent fascia and skin. The orbital portion participates in firm eyelid closure and in the smile‐related movement that creates lateral canthal rhytids; the palpebral portion contributes to blinking and eyelid closure [2]. For this reason, weakening the wrong portion may improve the line incompletely while disturbing normal eyelid function.

This principle is consistent with the MRI‐based work by Rams et al. [5], who examined the relationship between visible glabellar contraction patterns and underlying glabellar muscle anatomy. Although their study addressed the glabellar region rather than lateral canthal lines, it is conceptually relevant because it showed that visible surface contraction patterns should not be assumed to directly represent deeper muscle anatomy. By analogy, lateral canthal wrinkle morphology should be interpreted as a clinical surface sign rather than as a precise map of the orbicularis oculi muscle. This supports an anatomy‐first approach in which the injector evaluates the responsible muscle field, contraction vector, and adjacent functional structures instead of treating the visible crease as the injection target.

A useful bedside approach is to assess the patient at rest, in gentle smile, in maximum smile, and in squint. Dynamic lines that appear only with animation are more toxin‐responsive; etched static lines may need skin‐directed modalities or filler‐type support rather than escalating toxin dose [6]. During animation, the injector should identify the vector and zone of maximal muscle recruitment, then plan points in the lateral orbicularis field rather than inside the eye‐side end of the crease. The question is not “where is the wrinkle?” but “which muscle fibers are producing this wrinkle, and where can they be weakened without harming eyelid closure or smile?”

**FIGURE 3:**
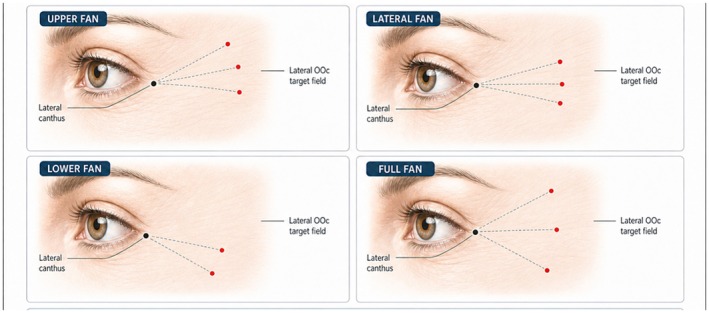
Pattern‐based planning for crow's feet. Schematic upper, lateral, lower, and full fan patterns. The figure emphasizes that injection points should be individualized according to the patient's animated line pattern, ocular risk factors, product, dose, and depth rather than placed mechanically into every visible crease.

Because crow's feet may present as upper, lower, lateral, or full fan patterns, patient‐specific planning is more defensible than placing toxin mechanically into every visible line [7, 8] (Figure 3).

## 3 The Problem With Injecting Directly at the Lateral Canthus

Injecting directly at the lateral canthus, or immediately on the wrinkle closest to the canthal angle, creates several problems. The first is insufficient efficacy. A tiny intradermal deposit over the crease may lie medial to the main lateral muscle belly, especially in a patient whose lateral orbicularis oculi is thick and strongly recruited. The treatment then softens the skin crease only indirectly, if at all, while leaving the stronger lateral contraction intact. The injector may respond by increasing the dose or volume, but a higher or more dilute injection can increase spread and make the treatment less controlled [9, 10, 11].

The second problem is the risk of diffusion to structures that should not be significantly weakened. The palpebral portion of the orbicularis oculi is essential for blinking and tear‐film distribution. Excessive weakening close to the eyelid can contribute to lagophthalmos, exposure symptoms, lower‐eyelid laxity, or dry‐eye complaints in susceptible patients. Ophthalmic literature and recent ocular‐surface data suggest that lateral canthal botulinum toxin can temporarily affect tear‐film stability, meibomian gland function, or ocular surface comfort, particularly when eyelid dynamics are disrupted [12, 13].

The third problem is diplopia and ptosis, rare but important adverse outcomes. Anatomical reviews warn that toxin placed close to the orbital rim or in excessive dose may diffuse toward extraocular muscles or the levator palpebrae superioris, producing diplopia or eyelid ptosis [2, 14, 15]. The problem is not that standard crow's feet treatment is unsafe when performed properly; large randomized trials and reviews support the efficacy and general tolerability of approved onabotulinumtoxinA patterns [16, 17, 18, 19]. The problem is that a line‐chasing technique may drift away from the safety logic of those patterns.

A fourth problem is smile asymmetry. If the inferior crow's‐foot is treated too low, too deep, or too heavily, diffusion may weaken the zygomaticus major, zygomaticus minor, or related midface elevators. The result may be less a smoother eye and more an asymmetric smile. The anatomical proposal by previous authors specifically reduced the dose at the inferior point because toxin spread in this region can affect zygomaticus muscles and facial expression [2]. This is a good example of muscle‐based thinking: the lower line is seen near the eye, but its treatment must respect the cheek smile complex.

## 4 Why a More Lateral Target Is Rational but Not Unlimited

A more lateral target can be anatomically rational because the active orbicularis oculi muscle often extends well lateral to the canthus. The lateral orbital rim at the level of the lateral canthus is a useful landmark, and recent anatomical guidance proposed injection points around the border of the orbicularis oculi, approximately 20 mm from the lateral orbital rim in superior, lateral, and inferior directions, with lower dosing at the inferior point [2]. Ultrasonography supports the concept that the lateral part of the orbicularis oculi is an important dynamic treatment zone [2, 3]. However, the current evidence supporting more lateral anatomy‐based placement is mainly anatomical and ultrasonographic. Comparative clinical studies demonstrating superior efficacy or safety over established injection patterns remain limited. Therefore, the proposed lateral placement should be interpreted as an anatomy‐based rationale for individualized treatment planning, not as proven clinical superiority or as a universal rule.

The difference is clinically important. “More lateral” should not be interpreted as a blind rule. Too posterior a point may be ineffective if it is outside the active muscle. Too inferior a point may approach the zygomaticus complex. Too deep a point may increase bruising or affect structures beyond the superficial orbicularis plane. The aim is not to escape the canthus by distance alone, but to translate the visible line into a safe neuromuscular target. A laterally placed, superficial, low‐volume injection in the active orbicularis field is conceptually different from a deep injection into the lateral cheek simply because the wrinkle points in that direction.

Dose economy is part of this logic. The Global Aesthetics Consensus emphasized diagnostic analysis of target muscles in relation to adjacent soft and hard tissues, with a shift toward neuromodulation rather than crude paralysis [20]. Consensus recommendations for modern botulinum toxin preparations also stress assessment, planning, patient education, and product‐specific dosing rather than generic transfer of units [21]. In a small periocular field, a conservative first treatment is often wiser than treating every line aggressively. The intended endpoint is softening of dynamic contraction while retaining blink, eye shape, and expression.

## 5 The Bunny Line Analogy

The bunny line is a helpful analogy because it makes the same anatomical error easier to see. Bunny lines appear as oblique or vertical creases near the nasal dorsum. Traditionally, they have often been treated by injecting the visible wrinkle under the nasal bridge. Recent cadaveric and ultrasonographic work showed that the area under many bunny lines is a nonmuscular “bunny triangle” located between the borders of the procerus, nasalis, orbicularis oculi, and levator labii superioris alaeque nasi muscles [4]. In that setting, a toxin injection placed directly into the wrinkle may be entering connective tissue rather than the muscle that produced the skin fold.

The lesson is not that the anatomy of bunny lines and crow's feet is identical. The lesson is that wrinkles can be displaced signs. A crease can overlie skin, septa, fascia, or connective tissue while the causative muscle lies at the border. In bunny lines, the treatment concept shifts from injecting the crease to targeting the neighboring muscular borders. In lateral canthal rhytids, the same philosophy should discourage injections directly into the canthal corner when the active orbicularis field lies lateral to it. The wrinkle is the map, but not always the destination.

This analogy also helps patients understand why an injector may mark a point away from the deepest line. To a patient, it may look as though the doctor is not treating the wrinkle. In fact, the doctor is treating the muscle that makes the wrinkle. A good consent discussion can explain that botulinum toxin is not filler, resurfacing, or scar revision. It is neuromodulation. The target is the contraction mechanism, and the skin improves because the contraction relaxes.

## 6 Practical Clinical Implications

First, evaluate before marking. Ask the patient to smile naturally, smile maximally, squint, and relax. Observe whether the line pattern is upper, lower, lateral, or full; whether the lower eyelid is lax; whether there is pre‐existing dry eye, tearing, prior eyelid surgery, thyroid eye disease, contact‐lens intolerance, or facial nerve weakness; and whether static etched lines dominate. These observations should influence point selection and dose. Treating a patient with lower‐lid laxity or dry eye exactly like a patient with a firm eyelid and only dynamic lines is not anatomically or clinically sound.

Second, mark landmarks before marking toxin points. The lateral canthus, lateral orbital rim, lower orbital rim, and cheek smile elevators should be understood in three dimensions. The lateral canthus helps orient the injector, but the planned injection may belong in the lateral orbicularis oculi field rather than directly at the canthal angle, depending on the patient's contraction pattern and risk profile. The product label and anatomical literature both support deliberate spacing from the eye and rim [1, 2]. However, lateral placement should be understood as an individualized anatomical strategy rather than a fixed rule or a proven superior protocol. Marking landmarks also reduces the temptation to follow every small crease, especially after the patient smiles and the skin becomes visually busy.

Third, use superficial placement and small aliquots when appropriate to the product and indication. The orbicularis oculi is superficial, and anatomical work recommends subdermal or intradermal approaches in lateral canthal rhytids, while also warning about bruising from periocular veins [2]. Diffusion is influenced by dose, concentration, injection volume, and technique [9, 10, 11]. A conservative, accurately placed injection is more rational than a larger volume meant to compensate for uncertain targeting.

Fourth, respect the inferior point. Lower crow's feet are common and cosmetically important, but the inferior periorbital‐cheek junction is where eyelid closure, tear film, and smile mechanics converge. A smaller dose, more lateral placement, or omission of the inferior point may be appropriate in selected patients. This is especially relevant when the lower line is shallow, when the patient already has dry eye or lower‐lid laxity, or when a strong zygomaticus smile is close to the proposed point. The best treatment may be an incomplete‐looking pattern on the diagram that is actually more complete for that patient's anatomy.

Fifth, document the reason for individualized placement. Many complications occur not because the toxin was used, but because the injector ignored anatomy, pattern, and dose. A simple note describing the patient's pattern, the rationale for conservative inferior dosing, and any ocular risk factors improves continuity of care. It also reminds the injector at follow‐up that a partial response may be corrected by anatomical adjustment, not automatically by a higher dose.

## 7 Follow‐Up: Read the Result Anatomically

Follow‐up is another opportunity to avoid line‐chasing. At review, the injector should compare rest and maximum‐smile images, not simply inspect the deepest remaining crease. If the lateral muscle is still strongly contracting, a small anatomical adjustment may be reasonable; if the dynamic contraction is controlled but an etched line remains, more toxin may not be the answer. Static dermal lines, photodamage, and skin laxity should be treated as skin and soft‐tissue problems rather than as evidence that the orbicularis oculi has not been weakened enough.

An incomplete result can also teach the injector where the true muscle vector was located. Residual upper fan lines may suggest undertreatment of the superior lateral orbicularis field; residual lower lines may require caution rather than automatic dose escalation, especially if the patient has lower‐lid laxity, dry eye symptoms, or a smile that depends heavily on nearby zygomaticus activity. In this sense, the follow‐up visit is a map‐reading exercise. The line tells the clinician where the contraction persisted, but the next injection still has to be planned from anatomy.

**FIGURE 4:**
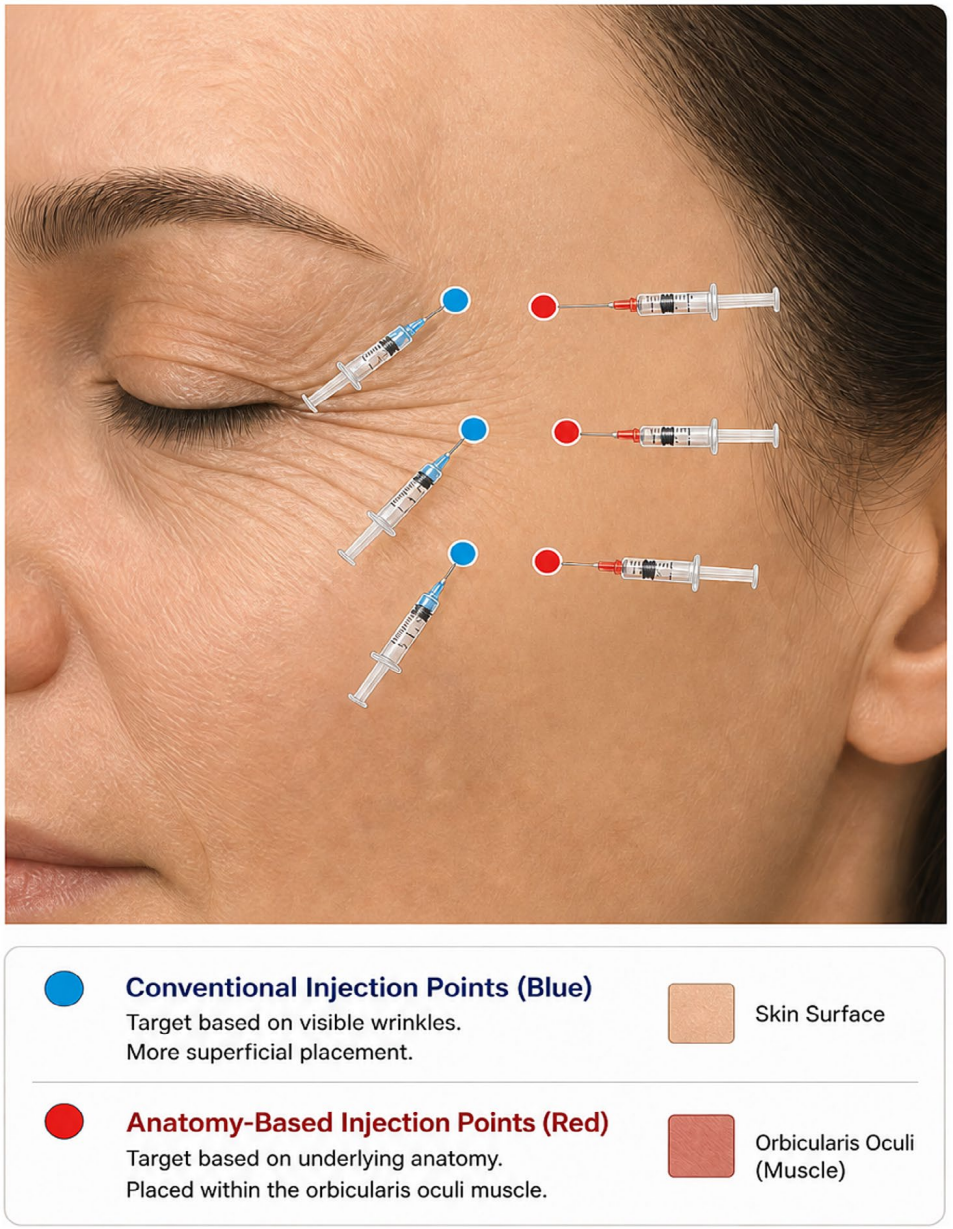
Conceptual comparison between wrinkle‐based and anatomy‐based injection planning for lateral canthal lines. Blue dots indicate conventional points selected primarily according to visible wrinkle location. Red dots indicate proposed anatomy‐based points placed more laterally within the orbicularis oculi muscle field. This figure illustrates an anatomical rationale for individualized planning and should not be interpreted as evidence of clinical superiority over established injection patterns. Further comparative clinical studies are required to determine relative efficacy and safety.

The safest correction is usually conservative and product‐specific. The injector should avoid rapid repeated treatment, excessive total dose, and high‐volume diffusion‐based corrections because the periocular region has little tolerance for unintended spread [9, 10, 11]. A standardized diagram is useful for documentation, but it should be accompanied by a written rationale for any point that was moved laterally, omitted, or reduced. That rationale is the sign that the treatment was based on the patient, not on the wrinkle alone (Figure 4).

## 8 Conclusion

The central problem with injecting botulinum toxin on the lateral canthus or directly on the lateral canthal wrinkle is that the visible crease is being mistaken for the therapeutic target. The crease is only the surface sign of a deeper dynamic system. In crow's feet, the responsible system is primarily the lateral orbital orbicularis oculi, with variable bands, periorbital tethering, eyelid function, and midface smile muscles. A canthal or line‐chasing injection may miss the thicker lateral muscle belly, overweaken palpebral orbicularis, diffuse toward orbital or extraocular structures, disturb tear‐film dynamics, or affect the zygomaticus complex.

A better clinical phrase is: target the muscle field, not the wrinkle. This does not mean injecting indiscriminately farther laterally, nor does it imply that anatomy‐based lateral placement has been proven clinically superior to established injection patterns. Rather, it means using the lateral canthus as a landmark, identifying the active contraction pattern, selecting safe superficial points in the lateral orbicularis oculi, and adjusting dose and distribution to the patient's line pattern and ocular risk. The bunny line analogy reinforces the same principle: a wrinkle may overlie nonmuscular tissue, while the effective target lies at the muscular border. In aesthetic neuromodulation, anatomy should guide the treatment plan, while clinical outcomes should continue to be validated in comparative studies.

## 9 Clinical Pearls

- Do not inject the deepest line automatically; translate the line into the responsible muscle field.
- Treat the lateral canthus as a landmark. In selected patients, the therapeutic target may lie within the lateral orbital orbicularis oculi field rather than directly at the canthal corner.
- Assess the pattern during animation and individualize upper, lateral, and lower points.
- Use conservative dose and volume around the eye, especially near the inferior point and in patients with ocular surface symptoms.
- Interpret anatomy‐based lateral placement as a rationale for individualized planning, not as proven superiority over established injection patterns.
- When response is incomplete, reconsider anatomy before simply increasing dose.

## Author Contributions

Conceptualization: Kyu‐Ho Yi. Writing – original draft preparation: Kyu‐Ho Yi, Kim Hongeok. Writing – review and editing: Kyu‐Ho Yi, Kim Hongseok. Visualization: Kyu‐Ho Yi, Kim Hongeok. Supervision: Kyu‐Ho Yi.

## Funding

The authors have nothing to report.

## Ethics Statement

The authors have nothing to report.

## Consent

The authors have nothing to report.

## Conflicts of Interest

The authors declare no conflicts of interest.

## Data Availability

The data that support the findings of this study are available from the corresponding author upon reasonable request.
